# The Assimilation of Diazotroph-Derived Nitrogen by Scleractinian Corals Depends on Their Metabolic Status

**DOI:** 10.1128/mBio.02058-16

**Published:** 2017-01-10

**Authors:** Vanessa N. Bednarz, Renaud Grover, Jean-François Maguer, Maoz Fine, Christine Ferrier-Pagès

**Affiliations:** a Marine Department, Centre Scientifique de Monaco, Monaco, Principality of Monaco; b LEMAR-UMR 6539 UBO/CNRS/IRD, Institut Universitaire Européen de la Mer, Plouzané, France; c The Mina and Everard Goodman Faculty of Life Sciences, Bar-Ilan University, Ramat-Gan, Israel; d The Interuniversity Institute for Marine Science, Eilat, Israel; University of Southern California

## Abstract

Tropical corals are associated with a diverse community of dinitrogen (N_2_)-fixing prokaryotes (diazotrophs) providing the coral an additional source of bioavailable nitrogen (N) in oligotrophic waters. The overall activity of these diazotrophs changes depending on the current environmental conditions, but to what extent it affects the assimilation of diazotroph-derived N (DDN) by corals is still unknown. Here, in a series of ^15^N_2_ tracer experiments, we directly quantified DDN assimilation by scleractinian corals from the Red Sea exposed to different environmental conditions. We show that DDN assimilation strongly varied with the corals’ metabolic status or with phosphate availability in the water. The very autotrophic shallow-water (~5 m) corals showed low or no DDN assimilation, which significantly increased under elevated phosphate availability (3 µM). Corals that depended more on heterotrophy (i.e., bleached and deep-water [~45 m] corals) assimilated significantly more DDN, which contributed up to 15% of the corals’ N demand (compared to 1% in shallow corals). Furthermore, we demonstrate that a substantial part of the DDN assimilated by deep corals was likely obtained from heterotrophic feeding on fixed N compounds and/or diazotrophic cells in the mucus. Conversely, in shallow corals, the net release of mucus, rich in organic carbon compounds, likely enhanced diazotroph abundance and activity and thereby the release of fixed N to the pelagic and benthic reef community. Overall, our results suggest that DDN assimilation by corals varies according to the environmental conditions and is likely linked to the capacity of the coral to acquire nutrients from seawater.

## IMPORTANCE

Tropical corals are associated with specialized bacteria (i.e., diazotrophs) able to transform dinitrogen (N_2_) gas into a bioavailable form of nitrogen, but how much of this diazotroph-derived nitrogen (DDN) is assimilated by corals under different environmental conditions is still unknown. Here, we used ^15^N_2_ labeling to trace the fate of DDN within the coral symbiosis. We show that DDN is assimilated by both the animal host and the endosymbiotic algae. In addition, the amount of assimilated DDN was significantly greater in mesophotic, bleached, or phosphorus-enriched corals than in surface corals, which almost did not take up this nitrogen form. DDN can thus be of particular importance for the nutrient budget of corals whenever they are limited by the availability of other forms of dissolved nutrients.

## INTRODUCTION

Corals are multipartite symbiotic organisms (holobionts) formed by the coral host and a diverse microbiota consisting of endosymbiotic dinoflagellates, endolithic algae, bacteria, archaea, fungi, and viruses (1, 2). The associated microbial community is specific to both the coral species and the microhabitat within the coral host (i.e., surface mucus layer, coral tissue, and coral skeleton); this highly diverse coral microbiome provides important services to the coral holobiont, such as nutrient acquisition and recycling or antimicrobial defense, among many others (3–5).

Nutrient, especially nitrogen (N), acquisition is of particular importance for corals thriving in oligotrophic reef waters, where N availability often limits primary productivity (6). Corals have therefore evolved an efficient internal recycling of N between the coral host and its dinoflagellate symbionts (7). Model calculations suggest that the symbiotic dinoflagellates can acquire up to 80% of their N from the animal host, indicating that recycling of the internal N pool plays a key role in holobiont functioning (8). Nevertheless, corals also require external N resources to match carbon (C) uptake and sustain net growth, which is gained via heterotrophic consumption of plankton by the coral host (9, 10), via dissolved inorganic N uptake by the dinoflagellates (11), as well as via the association with dinitrogen (N_2_)-fixing microbes (diazotrophs). A wide variety of diazotrophs has been discovered within the surface mucus layer, or the coral tissue, and skeleton, each harboring a distinct diazotrophic community (12, 13). Studies that used the natural δ^15^N signature of coral tissue demonstrated qualitatively that diazotroph-derived N (DDN) was assimilated by the coral when the δ^15^N signature of the coral was close to the δ^15^N value (0‰) of atmospheric N_2_ (14, 15). Other studies have quantitatively measured the N_2_ fixation activity of coral-associated diazotrophs (15–22). Most of these studies used the acetylene reduction assay that measures the nitrogenase activity of diazotrophs indirectly (15–19, 21). However, this approach does not provide quantitative insight into the net assimilation of DDN by the coral host or dinoflagellates. Instead, the use of labeled ^15^N_2_ gas measures net N_2_ fixation directly, thus allowing tracing of the fate and assimilation of DDN within the different coral compartments. So far, only two studies have applied the ^15^N_2_ technique to tropical scleractinian coral species (22, 23). The first study, which used the gas bubble method to enrich seawater with ^15^N_2_, found ^15^N enrichment only in the suspended particles of the incubation medium (i.e., particles released by the coral) and not within the coral tissue or dinoflagellates (22). Heterotrophic diazotrophs were thus mainly active in the coral-surrounding seawater, likely benefiting from the high availability of organic C contained in the mucus and released by corals during incubation (24). On the contrary, the second study used the ^15^N_2_-enriched seawater addition method and was able to trace DDN assimilation by the dinoflagellates (23). It also experimentally showed that corals could obtain DDN through the ingestion (heterotrophic feeding) of planktonic diazotrophs. The difference between the two studies might be due to the different methods used (^15^N_2_ gas bubble versus ^15^N_2_-enriched seawater addition), as it was suggested that the gas bubble method could, in some cases, underestimate N_2_ fixation rates (25, 26). In addition, other environmental factors, such as the diazotroph community composition, the corals’ metabolic status, or the N availability in the environment, may have also been involved.

The corals’ metabolic status (i.e., autotrophic, heterotrophic) is one of the main factors affecting both the rates of mucus release and the rates of organic particle uptake from the surrounding seawater (27). For example, heterotrophy will be high when the autotrophic C input by the symbiotic dinoflagellates is insufficient (i.e., bleached or deep corals [28, 29]). This might subsequently influence the coral’s DDN assimilation since heterotrophy is also linked to the predation of diazotroph particles (23). Both the corals’ metabolic status and the overall activity of coral-associated diazotrophs strongly depend on the current environmental conditions (17, 19, 29, 30), but to the best of our knowledge, no study has directly quantified the assimilation of DDN by corals under different environmental conditions so far. Furthermore, as coral mucus contains a high number of diazotrophs and corals are known to take up diazotrophs from the surrounding seawater (23, 31), the specific role of mucus in providing new bioavailable N to the coral host under different environmental conditions requires further investigation.

Therefore, the present study applied the ^15^N_2_-enriched seawater addition method in a series of incubation experiments with scleractinian corals from the northern Red Sea to better understand the importance of DDN assimilation for the coral holobiont. The aims were (i) to quantify DDN assimilation by corals (i.e., animal tissue and dinoflagellates) with different metabolic statuses, i.e., exposed to different environmental conditions (i.e., shallow, deep, bleached, and phosphate-enriched corals) and (ii) to investigate mucus-associated net N_2_ fixation and its potential contribution to the DDN assimilation by corals.

## RESULTS

### N_2_ fixation by suspended particles.

**FIG 1:**
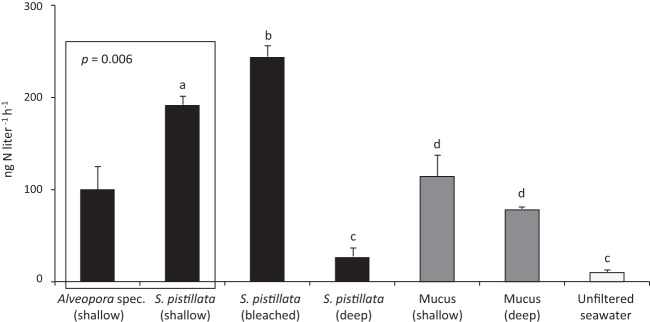
N_2_ fixation by suspended particles (ng N liter^−1^ h^−1^; mean ± SE) after incubation of *Alveopora* species (shallow) and *S. pistillata* (shallow, bleached, and deep) coral colonies (black bars), of coral mucus collected from shallow and deep *S. pistillata* (gray bars), and of unfiltered seawater samples (white bar). Different letters (a to d) above the bars indicate significant differences between the incubations (one-factor PERMANOVA with pairwise MC tests; significance level, *P* < 0.05). A separate PERMANOVA was conducted to compare N_2_ fixation by suspended particles between *Alveopora* species (shallow) and *S. pistillata* (shallow) incubations.

All ^15^N_2_ incubation experiments resulted in a significant ^15^N excess enrichment of the suspended particles compared to control samples, to which no ^15^N tracer was added (Table 1). N_2_ fixation by suspended particles (ng N liter^−1^ h^−1^, Fig. 1) was significantly different among the coral, mucus, and unfiltered seawater incubations (permutational multivariate analysis of variance [PERMANOVA]: pseudo-*F*^5,27^ = 42.11, Monte Carlo [MC] test *P* < 0.001). The lowest N_2_ fixation rates were observed in the unfiltered seawater control containing the natural community of planktonic diazotrophs (9.80 ± 1.90 ng N liter^−1^ h^−1^). As a reminder, in all of the other incubations, the natural community of planktonic diazotrophs was excluded by seawater filtration before the incubations and only the activity of diazotrophs that were associated with the coral mucus or had been expelled by the coral was measured. In this case, suspended particles released during coral incubations [except those with *Stylophora pistillata* (deep); 26.29 ± 8.96 ng N liter^−1^ h^−1^; pairwise MC tests, *P* > 0.05] and during mucus incubations (deep or shallow) showed significantly enhanced N_2_ fixation rates compared to natural planktonic diazotrophs of the unfiltered seawater control (pairwise MC tests, *P* < 0.05, Fig. 1). Highest N_2_ fixation by suspended particles was observed after incubation of *S. pistillata* (bleached) with 324.64 ± 64.55 ng N liter^−1^ h^−1^. The separate comparison of *Alveopora* species (shallow) and *S. pistillata* (shallow) revealed significant differences as well (PERMANOVA: pseudo-*F*^1,8^ = 19.08, *P* [MC] = 0.006).

**TABLE 1:** ^15^N excess enrichment of suspended particles, animal tissue, and dinoflagellates exposed to different environmental conditions^a^

Incubated sample	^15^N excess enrichment (atom% ^15^N_excess_)		
	Suspended particles	Animal tissue	Dinoflagellates
Coral			
* Alveopora* species (shallow)	0.121 ± 0.017^b^	0.0002 ± 0.0002	0.0017 ± 0.0002
* S. pistillata* (shallow)	0.349 ± 0.069^b^	0.0002 ± 0.0001	0.0014 ± 0.0002
* S. pistillata* (72 h)	0.315 ± 0.058^b^	0.0020 ± 0.0001^b^	0.0032 ± 0.0001^b^
* S. pistillata* (phosphate)	n.s.^c^	0.0021 ± 0.0001^b^	0.0043 ± 0.0002^b^
* S. pistillata* (bleached)	0.245 ± 0.105^b^	0.0033 ± 0.0003^b^	0.0059 ± 0.0032^b^
* S. pistillata* (deep)	0.083 ± 0.034^b^	0.0043 ± 0.0003^b^	0.0069 ± 0.0006^b^
Mucus			
Mucus (shallow)	0.072 ± 0.008^b^		
Mucus (deep)	0.058 ± 0.003^b^		
Unfiltered seawater	0.127 ± 0.009^b^		

a The data are mean ± SE. The ^15^N excess enrichment was calculated from the difference in atom% ^15^N between control samples (not exposed to ^15^N_2_) and samples after ^15^N_2_ exposure.

b Significant enrichment (at least three times as great as the standard deviation of control samples).

c n.s., not sampled.

**FIG 2:**
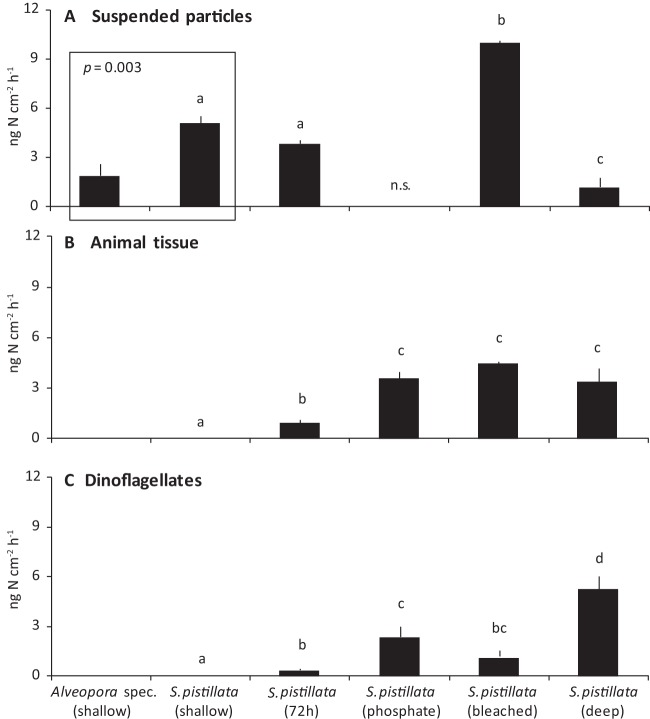
N_2_ fixation by suspended particles (A), as well as DDN assimilation by animal tissue (B) and dinoflagellates (C) after incubation of *Alveopora* species (shallow) and *S. pistillata* (shallow, 72 h, phosphate, bleached, and deep) coral colonies (ng N cm^−2^ h^−1^; mean ± SE). The suspended particles from the *S. pistillata* (phosphate) incubation were not sampled (n.s.) for analysis. Different letters (a to d) above the bars indicate significant differences between the *S. pistillata* incubations (one-factor PERMANOVAs with pairwise MC tests; significance level, *P* < 0.05). A separate PERMANOVA was conducted to compare N_2_ fixation by suspended particles between *Alveopora* species (shallow) and *S. pistillata* (shallow) corals (no significant ^15^N excess enrichment, and thus no DDN assimilation, was detected in the animal tissue and dinoflagellates of these corals).

When N_2_ fixation by suspended particles was normalized to the coral surface area (ng N cm^−2^ h^−1^, Fig. 2A), it showed significant differences between the *S. pistillata* incubations (PERMANOVA: pseudo-*F*^3,15^ = 63.62, *P* [MC] < 0.001), with the highest rates during the *S. pistillata* (bleached) incubation and the lowest during the *S. pistillata* (deep) incubation. Also the comparison of *Alveopora* species (shallow) and *S. pistillata* (shallow) revealed significant differences (PERMANOVA: pseudo-*F*^1,8^ = 19.08, *P* [MC] = 0.003). Overall, both normalizations gave the same trends.

### DDN assimilation in coral tissue and dinoflagellates.

The ^15^N excess enrichment of the dinoflagellates always exceeded that of the animal tissue, but in both coral compartments, it was 10- to 100-fold lower than the enrichment of the suspended particles (Table 1). Compared to the coral controls (no ^15^N_2_ exposure), we detected a significant ^15^N excess enrichment of the coral tissue and dinoflagellates in all ^15^N_2_ incubation experiments, except for *Alveopora* species (shallow) and *S. pistillata* (shallow). Although we detected no ^15^N excess enrichment of *S. pistillata* (shallow) after an incubation period of 24 h, the animal tissue and dinoflagellates were significantly enriched after repeated incubation of the corals in ^15^N_2_-enriched seawater over 3 consecutive days [*S. pistillata* (72 h)], indicating slow but continuous DDN assimilation by the corals. Taking the N content of the animal tissue and dinoflagellates into account, the net DDN assimilation into the two coral compartments was calculated (ng N cm^−2^ h^−1^). This showed significant differences in DDN assimilation by the corals exposed to different environmental conditions for both the tissue (PERMANOVA: pseudo-*F*^4,18^ = 79.08, *P* [MC] < 0.001, Fig. 2B) and the dinoflagellates (PERMANOVA: pseudo-*F*^4,20^ = 19.64, *P* [MC] < 0.001, Fig. 2C). A significant assimilation in the animal tissue and dinoflagellates was detected in all corals except again in *Alveopora* species (shallow) and *S. pistillata* (shallow), which showed no ^15^N excess enrichment. *S. pistillata* incubated for 72 h presented the lowest DDN assimilation, in both the animal tissue and dinoflagellates. Compared to *S. pistillata* (72 h), the assimilation significantly increased in the animal tissue of *S. pistillata* (phosphate), *S. pistillata* (bleached), and *S. pistillata* (deep), as well as in the dinoflagellates of *S. pistillata* (phosphate) and *S. pistillata* (deep). The highest assimilation was detected in the dinoflagellates of *S. pistillata* (deep).

### Total net N_2_ fixation.

The total net N_2_ fixation of the coral incubations represents the sum of N_2_ fixation by suspended particles (normalized to the coral surface area; ng N cm^−2^ h^−1^) and the amount of DDN assimilated into the animal tissue and dinoflagellates. It was significantly different between the *S. pistillata* incubations (PERMANOVA: pseudo-*F*^3,15^ = 35.30, *P* [MC] < 0.001), with the lowest rates for the shallow *S. pistillata* corals incubated for either 24 h (5.14 ± 0.38 ng N cm^−2^ h^−1^) or 72 h (5.21 ± 0.08 ng N cm^−2^ h^−1^). Compared to these corals, the total N_2_ fixation was ~3- and ~2-fold higher in bleached *S. pistillata* (15.67 ± 0.38 ng N cm^−2^ h^−1^) and deep *S. pistillata* (10.44 ± 1.26 ng N cm^−2^ h^−1^), respectively. Furthermore, *S. pistillata* (72 h) assimilated only ~25% of the total fixed N into the animal tissue and dinoflagellates, while the assimilation increased to ~35% in *S. pistillata* (bleached) and up to ~90% in *S. pistillata* (deep).

## DISCUSSION

Using ^15^N_2_ isotopic labeling, this study provides new insights into the assimilation of DDN by tropical corals exposed to different environmental conditions. The results indicate that DDN assimilation strongly varies with the autotrophic/heterotrophic status of the coral holobiont and with the phosphate availability in seawater, revealing an increased assimilation of DDN by corals that rely more on heterotrophy (i.e., bleached and deep corals) or are exposed to increased phosphate levels. Furthermore, we also demonstrate that mucus can be a major source of new N for the coral host (via feeding on diazotrophs contained in the mucus) and for the coral reef community (via the release of mucus, enriched in diazotrophs and fixed N compounds, to the surrounding seawater).

### N_2_ fixation by suspended particles.

N_2_ fixation by suspended particles quantified in unfiltered seawater samples (containing the natural planktonic diazotroph community) averages 9.80 ± 1.90 ng N liter^−1^ h^−1^. This rate is higher than those previously measured in other ^15^N_2_-labeling studies (<1.1 ng N liter^−1^ h^−1^) for seawater of the northern Gulf of Aqaba (32, 33) but is in the range of those measured (10.8 ± 0.6 ng N liter^−1^ h^−1^) in coastal waters of New Caledonia (34). N_2_ fixation rates vary significantly with the abundance and activity of diazotrophs, themselves depending on environmental factors such as temperature, depth, light, and nutrient and trace metal availability (35). Here we show that N_2_ fixation rates in coral mucus samples are 12 times as high as those in unfiltered natural seawater (without mucus). This is likely due to a high diazotroph abundance in the mucus, which can be up to 400-fold higher than that in the surrounding seawater (31). Coral mucus is rich in dissolved organic C and inorganic phosphate, which is required for bacterial growth (36, 37). Once released into seawater, it stimulates the activity of both mucus- and seawater-associated bacteria by providing a suitable energy and nutrient source (37–39). Thus, the increased N_2_ fixation observed in our mucus incubations could be the result of both the net release of diazotrophs into seawater and the stimulation of their activity. Our results are more pronounced than those of Camps et al. (31), who found only an insignificant 2-fold increase in N_2_ fixation when coral mucus was added to seawater samples. This may result from more active diazotrophs associated with the coral mucus in the present study. Those authors also measured up to 70-fold lower planktonic N_2_ fixation rates than previous measurements in the same area and season (34), also indicating that planktonic diazotrophs were less active.

During the coral incubations, suspended particles particularly released by the shallow corals showed up to 2-fold higher N_2_ fixation rates than suspended particles during the mucus-alone incubations (without corals). Corals continuously release approximately half of their photosynthetically acquired C as organic matter into the surrounding seawater (40, 41). This continuous supply of fresh mucus during coral incubations may have further increased the abundance of diazotrophs and stimulated their activity, while the availability of C was likely much reduced during the 24-h incubation with mucus alone. Mucus is indeed rapidly degraded (7 to 10% h^−1^) by bacteria (42), which, in particular, draw down its content in essential molecules such as phosphate and N (43). Our results are in agreement with the previous findings of Grover et al. (22), who also detected significant rates of N_2_ fixation by suspended particles released after the incubation of coral colonies, but the rates were much lower than those in the present study. The discrepancies between the two studies might be explained by the use of the ^15^N_2_ bubble rather than the ^15^N_2_-enriched seawater addition method, but also by seasonal variations (end of summer versus autumn) in diazotroph composition/activity, in the seawater nutrient concentrations, or in the N needs of the coral colonies, respectively. N_2_ fixation stimulated by corals is indeed highly variable on a seasonal scale, with the highest rates measured during the nutrient-depleted warm summer season (18, 19). We found the highest rates of N_2_ fixation by suspended particles after the incubation of *S. pistillata* (bleached). This may result from a higher abundance/activity of mucus-associated diazotrophs and may be linked to an increased release of labile mucus compounds by the coral (44). Interestingly, N_2_ fixation by suspended particles did not increase when a deep-water coral colony was incubated. Deep corals show reduced photosynthesis rates as a result of decreased light availability (45, 46). This may be accompanied by less C translocation from the symbiotic dinoflagellates to the host and reduced rates of organic matter release, as previously demonstrated in shallow-water corals exposed to reduced light levels (47, 48). Together, these processes may have affected the activity of diazotrophs, leading to decreased N_2_ fixation rates by suspended particles. In addition, deep corals rely more on heterotrophy because of reduced photosynthesis (46). Increased heterotrophic feeding on organic matter and on diazotrophs in the mucus may all have lowered the amount of fixed N in suspended particles. Also, we detected in deep corals a higher ^15^N enrichment in both the animal tissue and the dinoflagellates compared to shallow corals (see discussion below). This further supports the idea that deep corals may have assimilated more suspended particles (containing diazotrophs and fixed N) from seawater as a result of increased heterotrophic feeding that, in turn, lowered the amount of fixed N in the suspended particles. Overall, it was suggested that mucus-associated diazotrophs, once released into seawater, provide an additional source of N to the pelagic and benthic reef compartments (31). In addition, our results indicate that this input of N to the coral reef may strongly differ between shallow and deep reef habitats, in relation to the autotrophic or heterotrophic status of corals.

### DDN assimilation by shallow-water corals.

All corals assimilated DDN into the animal tissue and the dinoflagellates, except for *Alveopora* species (shallow) and *S. pistillata* (shallow). This confirms the first observations of Grover et al. (22) on tropical corals that, under some conditions, corals do not assimilate fixed N, although this additional N source would be particularly beneficial for shallow corals. Shallow corals receive photosynthetically derived C in excess and are thus N rather than C limited. To cope with this N deficiency, shallow corals have an efficient uptake of dissolved inorganic N from seawater that is energetically fueled by high light availability (49; L. Ezzat and C. Ferrier-Pagès, unpublished data). Although we observed no DDN assimilation by shallow corals after 24 h, *S. pistillata* (72 h) assimilated approximately 30% of the total fixed N into the animal tissue (0.97 ± 0.14 ng N cm^−2^ h^−1^) and dinoflagellates (0.39 ± 0.08 ng N cm^−2^ h^−1^) after an incubation period of 72 h. This suggests that assimilation of DDN by shallow corals is a slow process and that N_2_ fixation is associated with the released coral mucus rather than with the corals, at least in shallow reef environments. A recent study using the ^15^N_2_-enriched seawater addition method on shallow *S. pistillata* corals from New Caledonia detected DDN assimilation exclusively in the dinoflagellates (~330 ng N cm^−2^ h^−1^) with assimilation rates up to 3 orders of magnitude higher than in the present study (23). Although both studies used the same incubation technique, different ^15^N_2_ enrichment percentages (10% versus 20%) at the start of incubation and different incubation times (4 h versus 24 h) may have affected both the diazotrophic activity and the transfer of DDN into the different coral compartments. Dinoflagellates assimilate ammonium (the end product of N_2_ fixation) much faster than the coral host (50). Also, Kopp et al. (51) observed a time lag of 6 h between N assimilation by the dinoflagellates and subsequent N translocation to the coral host, suggesting that incubation periods of >4 h are necessary to detect DDN assimilation in the animal tissue. Also, *S. pistillata* from the Red Sea may be associated with a diazotroph community that is different from or less active than that of corals from New Caledonia. Last, N_2_ fixation, as well as the assimilation of dissolved inorganic N from the surrounding seawater, can strongly vary with the current environmental conditions (19, 49, 52) and can be a reason for the observed differences. In the following, we provide new insights into DDN assimilation by shallow-water corals exposed to increased phosphate levels in seawater, as well as after bleaching.

### (i) Effect of phosphate availability.

Phosphate availability in seawater can positively affect the N_2_ fixation activity of planktonic diazotrophs (53). The high phosphorus demand for N_2_ fixation can be explained by the high ATP requirements for nitrogenase function, as previously demonstrated on plants (54, 55). This phosphorus constraint can occur either because fixation uniquely requires phosphorus-rich metabolites (56–58) or because fixation allows fixers to grow and thus creates phosphorus demand (59). In addition, it was previously shown for *S. pistillata* that ammonium uptake from seawater increases up to 2.5-fold in the presence of 0.5 or 3.0 μM phosphate because one nutrient is limiting the use of the other (52). Since ammonium is the final product of N_2_ fixation, it may explain the up to 5-fold higher DDN assimilation by the coral holobiont when it is exposed to pulses of 3 μM phosphate. Also, N_2_ fixation can be significantly reduced under low-phosphate conditions (60), and background phosphate levels in the Gulf of Eilat are very low (<0.1 μM) throughout the year (32, 61). This low availability of phosphate in seawater may also explain the significant effect we found on N_2_ fixation rates upon exposure to 3 μM phosphate. We cannot calculate the total N_2_ fixation under increased phosphate availability because of the lack of quantification of ^15^N enrichment in the suspended particles released by the coral. However, total N_2_ fixation by *S. pistillata* (phosphate) likely increased since the amount of DDN assimilated in the animal tissue and dinoflagellate compartment of *S. pistillata* (phosphate) already exceeded the total N_2_ fixation by *S. pistillata* (shallow).

### (ii) Effect of bleaching.

In the present study, incubations of bleached coral colonies revealed the highest total N_2_ fixation. The N_2_ fixation by suspended particles in this experiment was twice as high as the N_2_ fixation by suspended particles in the *S. pistillata* (shallow) incubation. Also, the DDN assimilation into tissue and dinoflagellates was greatly increased by *S. pistillata* (bleached) similarly to what was found for *S. pistillata* (phosphate). This enhanced activity of total (net) N_2_ fixation is similar to what was recently found for overall (gross) N_2_ fixation activities in bleached corals with the acetylene reduction assay (30). An increased seawater temperature also increases the abundance and diversity of coral-associated diazotrophs, shifting toward a more heat-resistant diazotroph community (62). Also, the bacterial community associated with the mucus undergoes drastic changes when exposed to heat stress (63). In the present study, ^15^N_2_ incubations with *S. pistillata* (bleached) were conducted after the heat stress period under normal temperature conditions. Thus, increased activity of the nitrogenase enzyme due to higher temperature can be excluded, but the corals most likely experienced a shift in the diazotrophic community that could be one reason for the increased N_2_ fixation rates seen (62). Furthermore, decreased photosynthesis rates in bleached corals lead to reduced O_2_ levels in the animal tissue and mucus layer. This, in turn, favors the activity of the O_2_-sensitive nitrogenase enzyme and can result in increased N_2_ fixation rates (64). Decreased photosynthesis reduces not only the O_2_ levels around the corals but also the autotrophic C supply to the host. This makes the coral more dependent on heterotrophic food sources, although the heterotrophic capacity (i.e., grazing) in bleached corals was also found to be impaired. A shift from catching larger high-energy prey items (i.e., nanoflagellates) to smaller low-energy prey (i.e., bacteria and picoflagellates) was observed in bleached *S. pistillata* because of the reduced energy required for catching nonmotile bacteria (65). Consequently, increased grazing on bacteria from the surrounding water may explain the higher DDN assimilation into the tissue of bleached corals observed here. Moreover, net organic matter fluxes in *S. pistillata* (bleached) changed from net uptake toward net release likely to reduce the effects of photoinhibition or cell membrane disruption (65–67). Increased release of organic matter can provide additional energy to seawater and mucus-associated diazotrophs, subsequently stimulating their abundance and/or N_2_ fixation activity in the surrounding water. Overall, the results show not only that total N_2_ fixation increases in incubations with bleached versus nonbleached corals but also that bleached corals assimilate more DDN. To what extent this additional N is beneficial for the recovery of the coral host after a bleaching event needs further investigation.

### DDN assimilation by deep-water corals.

Overall, the total N_2_ fixation was ~2-fold higher in incubations of *S. pistillata* (deep) than in incubations of *S. pistillata* (shallow or 72 h). One explanation for the increased N_2_ fixation activity with depth may be reduced light availability (350 versus 20 μmol m^−2^ s^−1^) leading to reduced O_2_ production via photosynthesis in corals (46). On a diurnal scale, coral-associated N_2_ fixation was found to be highest during twilight, when O_2_ production is not too high to inhibit nitrogenase, the enzyme responsible for N_2_ fixation (68, 69), but high enough for corals to deliver photosynthetic energy to the diazotrophs (15). Thus, the diurnal window with optimal O_2_ levels for N_2_ fixation may be greatly increased with increasing water depth, leading to higher N_2_ fixation rates. Previously, the abundance of scleractinian corals (*Montastraea cavernosa*, Bahamas) associated with diazotrophs was found to correlate positively with increasing water depth (15). This suggests that the diazotrophic community composition associated with corals may vary with depth and that the assimilation of DDN by corals may increase with depth as well. Also, the natural δ^15^N isotopic signature of scleractinian corals tends to be depleted as depth increases (70, 71). This can result from the assimilation of isotopically depleted DDN or from the uptake of isotopically depleted nitrate derived from atmospheric deposition, as for the Gulf of Aqaba/Eilat (72, 73). Here we show not only that the total N_2_ fixation increased in the incubations with deep versus shallow corals but also that deep corals assimilated 80% (8.65 ng N cm^−2^ h^−1^) of the total fixed N into the animal tissue or dinoflagellates. In contrast, shallow corals assimilated only 0% or 30% (1.36 ng N cm^−2^ h^−1^) of the total fixed N when incubated for 24 or 72 h, respectively. The higher assimilation by deep corals may result from a shift in the heterotrophic status of the coral. Corals in mesophotic reef environments generally rely more on other trophic strategies (i.e., heterotrophy) than their shallow counterparts in order to meet their metabolic demand and to compensate for the reduced photosynthetic C supply from their symbiotic dinoflagellates (45, 74). We found that the DDN assimilation by shallow and deep corals is inversely correlated with the amount of fixed N quantified in suspended particles in the coral incubation. Furthermore, the amount of fixed N in suspended particles was much smaller after the incubation of deep coral colonies than after the incubation alone of mucus extracted from deep coral colonies. This indicates that the high DDN assimilation into the tissue and dinoflagellates of deep corals is derived mainly via increased heterotrophic feeding on suspended particles rich in fixed N and diazotrophs that have been associated with the mucus. Although deep corals are considered to be less N limited by receiving both C and N from heterotrophic sources, they assimilate more DDN than their shallow counterparts. This could be explained by the fact that deep corals are light limited not only for photosynthesis but also for the uptake of dissolved inorganic N (49; Ezzat and Ferrier-Pagès, unpublished). Therefore, the increased DDN assimilation by deep corals may help to compensate for the reduced inorganic N uptake and may be particularly beneficial during periods when organic particles in seawater are insufficient to fully cover their N demand. The ingestion of planktonic free-living diazotrophs can also provide a substantial amount of N to the coral host, even exceeding the N input from coral-associated diazotrophs (23). Thus, it will be interesting to also investigate the importance of planktonic free-living diazotrophs for the N budget of mesophotic corals.

### Contribution of DDN to the coral N budget.

**FIG 3:**
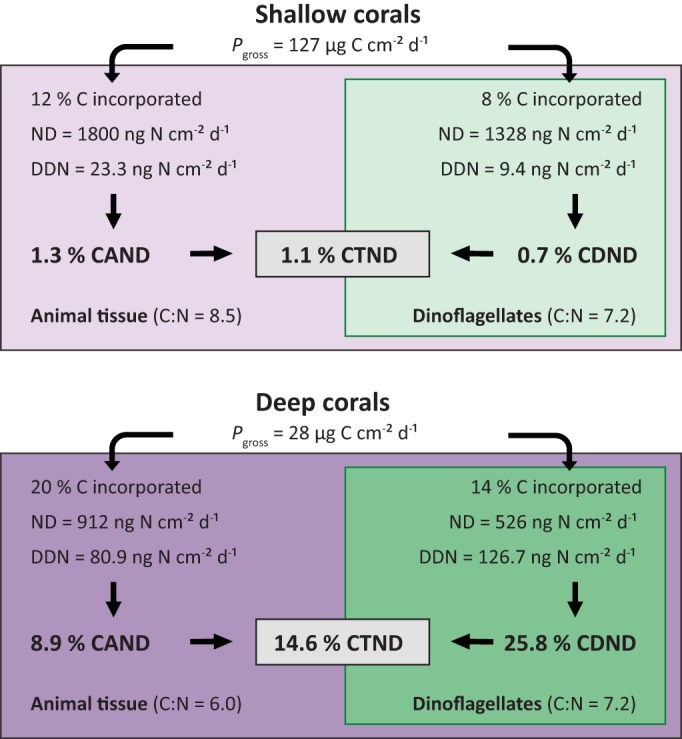
Overview of the percent contribution of DDN assimilation to the N demand (ND) of the animal host (CAND), the dinoflagellates (CDND), and the total symbiotic association (CTND) from shallow and deep *S. pistillata* corals. The ND was calculated from gross photosynthesis (*P*_gross_) measurements (45), from values of C incorporation into the animal host and the dinoflagellates (48), and from C/N ratios (70) of *S. pistillata* corals at 5- and 50-m water depths in the Red Sea.

Tropical corals are generally well adapted to flourish in nutrient-poor waters by efficiently recycling ammonium between the animal host and symbiotic dinoflagellates, by heterotrophic feeding on organic N sources, and by actively scavenging dissolved inorganic N from seawater even at very low dissolved N concentrations. In order to estimate the importance of DDN in relation to other inorganic N sources, we compared the present results with data on rates of inorganic N uptake by corals. The rate of ammonium uptake by *S. pistillata* at nearly natural ambient concentrations (0.2 μM) was ca. 30 ng N cm^−2^ h^−1^, taking into account the animal and dinoflagellate assimilation rates, except for highly fed corals, for which the uptake was lower (49). In addition, the total rate of nitrate uptake by *S. pistillata* at the same low concentrations was equal to 1.5 ng N cm^−2^ h^−1^ (75). Overall, dissolved inorganic N contributes for a mean of 30 to 32 ng N cm^−2^ h^−1^, although these rates are an approximation, as they vary with light or temperature levels, as well as with other physicochemical and biological factors. If we assume similar inorganic N uptake rates for all of the corals in the present study, DDN can contribute up to ~1.4 ng N cm^−2^ h^−1^ in shallow corals or ca. 5% of the inorganic N uptake. This contribution increases up to ~6 ng N cm^−2^ h^−1^ in bleached or phosphate-enriched corals and up to ~9.5 ng N cm^−2^ h^−1^ in deep corals, representing ca. 20 and 30% of the inorganic N uptake, respectively. In deep corals, with respect to their reduced inorganic N uptake capacity, this contribution is likely to be higher, underlining the importance of DDN for the N requirements of deep corals in relation to other inorganic N sources. As the ambient inorganic N concentrations on tropical coral reefs are often too low to sustain net growth of the coral holobiont (49), the association with diazotrophs likely serves as additional adaptation of corals to overcome N limitation in oligotrophic reef environments. In order to calculate the contribution of DDN to cover the N demand of deep and shallow corals, we used gross photosynthesis rates, C/N ratios, and assimilation rates of photosynthetically acquired C from previous studies with *S. pistillata* corals from the Red Sea exposed to high- and low-light conditions (45, 48, 70). This shows that DDN contributes only ~1% of the total N demand of shallow corals, while it can increase up to ~15% in deep corals (Fig. 3). Furthermore, in deep corals, DDN satisfies the N demand of dinoflagellates (~26%) to a greater extent than the N demand of the animal host (~9%), likely because of more efficient DDN assimilation by the former. Dinoflagellates can use N_2_ fixation products in the form of ammonium much faster than the coral host, as previously demonstrated for ammonium uptake from seawater (50). Instead, the coral host assimilates DDN after the synthesis of host macromolecules deriving from prey digestion or after translocation of recycled DDN by dinoflagellates (23). The higher contribution in deep corals is likely a product of both their higher DDN assimilation and their lower N demand, indicating that deep corals are likely less N limited than shallow corals (76). Overall, the present study shows that DDN can contribute importantly to the overall N budget of mesophotic corals, but whether this additional N is beneficial for the resistance and recovery of deep corals from environmental disturbances requires further investigation.

## MATERIALS AND METHODS

### Coral collection and experimental setup.

This study was conducted during November 2014 at the Interuniversity Institute for Marine Science (IUI), northern Gulf of Aqaba/Eilat. By using scuba gear, corals with fragment sizes with a 10- to 30-cm^2^ surface area were collected from the reef at both 5-m and 40- to 50-m water depths. While light conditions decreased from 200 to 400 μmol quanta m^−2^ s^−1^ at 5 m to 20 to 40 μmol quanta m^−2^ s^−1^ at 40 to 50 m, the seawater temperature (24.3 ± 0.1°C) and phosphate (338 ± 145 nM) and total dissolved inorganic N (ammonium, nitrite, and nitrate; 507 ± 131 nM) concentrations remained stable throughout the investigated depth range (mean ± standard error [SE]). Environmental data were provided by the Israel National Monitoring Program at the Gulf of Eilat (http://www.iui-eilat.ac.il/Research/NMPMeteoData.aspx) and represent average values collected during November 2014 at three coastal sampling stations (Taba, Japanese Gardens, Water Control Station) close to our study site.

In total, three different substrates (i.e., unfiltered seawater samples, coral fragments, and coral mucus samples) were collected for the following ^15^N_2_ incubation experiments (Table 2). Unfiltered seawater samples (without corals or mucus) were incubated in order to investigate net rates of N_2_ fixation by the natural planktonic diazotroph community (i.e., particles suspended in seawater). It must be noted that, except in incubations with unfiltered seawater, coral and coral mucus incubations were performed with 0.2-µm-filtered seawater. Therefore, all of the fixed N in the suspended particles of these incubations was derived from coral-associated diazotrophs (probably from bacteria within the mucus layer). For the mucus incubation, six large colonies of *S. pistillata* were collected from 5-m and 40- to 50-m depths, respectively. Mucus samples were extracted by gently spraying 500 ml of 0.2-µm-filtered seawater onto colonies maintained in air. This mucus mixture was subsequently incubated as described below to investigate rates of N_2_ fixation by mucus-associated diazotrophs in the absence of corals (i.e., particles suspended in mucus). Mucus samples collected from shallow and deep corals will be referred to as mucus (shallow) and mucus (deep), respectively.

**TABLE 2:** Summary of the different ^15^N_2_ incubation experiments conducted with coral fragments, coral mucus, and unfiltered seawater samples to quantify DDN assimilation and/or N_2_ fixation rates by the different incubated substrates

Incubated substrate	Depth (m)	Treatment before ^15^N_2_ incubation	^15^N_2_ incubation time (h)
Coral fragments^a^			
*Alveopora* species (shallow)	5	None	24
*S. pistillata* (shallow)	5	None	24
*S. pistillata* (72 h)	5	None	3 × 24
*S. pistillata* (phosphate)	5	3 μM H_2_PO_4_	24
*S. pistillata* (bleached)	5	Thermal-stress-induced bleaching	24
*S. pistillata* (deep)	40–50	None	24
Coral mucus^b^			
Mucus (shallow)	5	None	24
Mucus (deep)	40–50	None	24
Unfiltered seawater^c^	5	None	24

a DDN assimilation by animal tissue and dinoflagellates, N2 fixation by mucus and other suspended particles continuously released by corals.

b N_2_ fixation by mucus suspended particles.

c N_2_ fixation by seawater suspended particles (natural planktonic diazotrophs).

The coral incubations were used to quantify the assimilation of DDN by animal tissue and dinoflagellates and to investigate net rates of N_2_ fixation by suspended particles within the incubation medium in the presence of corals. Compared to the mucus incubation, the suspended particles in this experiment represent mucus and other particles continuously released by the coral. Three different sets of corals were collected (Table 2). (i) The first set represents shallow-water corals that were directly incubated (right after sampling) as described below without further treatment. Fragments of *Alveopora* species (*n* = 4) and *S. pistillata* (*n* = 5) were collected from a 5-m water depth. These fragments will be referred to *Alveopora* species (shallow) and *S. pistillata* (shallow). Additional *S. pistillata* fragments (*n* = 3) from a 5-m depth were incubated for 3 days to determine long-term DDN assimilation. In order not to incubate the corals in the same water for 72 h, the incubation water was renewed every 24 h and filtered for ^15^N analysis, while the coral fragments were only sampled for ^15^N analysis at the end of the 3 days. These fragments will be called *S. pistillata* (72 h). (ii) The second set represents shallow-water corals that were treated as described below prior to the ^15^N_2_ incubation experiments. Fragments of *S. pistillata* collected at a 5-m depth and transferred to six aquaria in outdoor water tables (natural light) with temperature and photosynthetic photon flux density (PFD) sets that corresponded to those found at 5 m. Ambient PFD was obtained by applying layers of black mesh above the tables. The ambient seawater temperature (24 to 25°C) was kept constant by using high rates of seawater renewal in the aquaria. For this set of corals, two treatments were applied in triplicate aquaria (with two fragments per aquarium). Three tanks maintained with a continuous seawater renewal rate of 20% h^−1^ were submitted to a thermal stress in which the temperature was gradually increased to 30°C for a week (1°C/day) with aquarium heaters connected to electronic controllers. After coral fragments visually bleached [here called *S. pistillata* (bleached)], the temperature was returned to 25°C within a week for the following incubation experiments. Three other tanks were maintained for 2 days in a closed circuit under elevated phosphate levels achieved by enriching the aquaria with a pulse of 3 µM H_2_PO_4_ twice a day [here called *S. pistillata* (phosphate)]. In the latter condition, the seawater was changed every day and the following incubations were conducted in phosphate-enriched seawater. (iii) The last set represents fragments of mesophotic *S. pistillata* (*n* = 5) collected from a 40- to 50-m depth and incubated directly after sampling. These fragments will be called *S. pistillata* (deep).

### ^15^N_2_ incubation experiments.

We used the ^15^N_2_-enriched seawater addition method and produced ^15^N_2_-enriched seawater prior to the incubation experiment by injection of 10 ml of ^15^N_2_ gas (98% Eurisotop) into degassed, 0.2-μm-filtered seawater, followed by vigorous shaking for at least 12 h. This procedure theoretically ensures 90 to 100% ^15^N_2_ equilibration (26). Afterward, each coral fragment was individually placed into a 500-ml gas-tight glass bottle completely filled with 450 ml of seawater (0.2 μm filtered) and 50 ml of ^15^N_2_-enriched seawater. Additional 500-ml bottles containing either 450 ml of the mucus mixture or 450 ml of the unfiltered seawater were incubated similarly after adding 50 ml of ^15^N_2_-enriched seawater. On the basis of the volume of the incubation bottle and the quantity of ^15^N_2_-enriched seawater added, this resulted in a theoretical enrichment of ~9.8 atom% ^15^N in the incubation medium of all bottles, similar to previous ^15^N_2_ studies of corals and sponges (22, 31, 77, 78). The addition of ^15^N_2_-enriched seawater can potentially contaminate the sample with trace metals that can affect N_2_ fixation rates, particularly in areas were trace metals are limiting (79). As the Gulf of Aqaba/Eilat is known to receive relatively large amounts of trace metals compared to other areas (80), a potential effect of contamination was considered to be insignificant. All bottles were closed gastight and incubated for 24 h [except for *Stylophora* (72 h)]. Additional coral, mucus mixture, and unfiltered seawater incubations without ^15^N_2_ addition served as control samples (^15^N background). During the incubations, bottles were continuously shaken in water baths equipped with agitator plates. Water baths were maintained under the right light and temperature conditions as described above by applying the same filters to shade the solar irradiance to the desired level and using temperature controllers.

At the end of the incubation, coral fragments were collected from the chambers, rinsed with filtered seawater to remove their mucus, and stored frozen until ^15^N analysis of the animal tissue and dinoflagellate fractions. The water of all incubations (coral, mucus, and unfiltered seawater) was filtered onto precombusted (400°C, 4 to 5 h) GF/F filters, and filters were also stored frozen until ^15^N analysis of suspended particles from the incubation water.

### Sample analysis.

The coral tissue was completely removed from the skeleton with an air brush and homogenized with a potter tissue grinder. Homogenates were then separated in several centrifugation steps into the animal and algal fractions according to reference 49, and each fraction was subsequently freeze-dried. All GF/F filters were dried in an oven (60°C, 48 h) prior to ^15^N analysis of the suspended particles. Finally, the organic N content together with its ^15^N enrichment in the animal tissue, dinoflagellates, and suspended particles was quantified with a mass spectrometer (Delta Plus; Thermo Fisher Scientific, Germany) coupled via a type III interface to a C/N analyzer (Flash EA; Thermo Fisher Scientific). The standard deviation obtained from repeated measurements of control samples was better than 0.5 µg for PN in the range used (5 to 50 µg N) and was <0.0001 atom% for ^15^N enrichment. The ^15^N excess enrichment for each sample was calculated with the formula atom% ^15^N_excess_ = (atom% ^15^N_sample _− atom% ^15^N_contro*l*_), where atom% ^15^N_sample_ is the ^15^N enrichment of samples after exposure to ^15^N_2_-enriched seawater and atom% ^15^N_control_ is the natural ^15^N enrichment of control samples without ^15^N_2_ exposure. The atom% ^15^N_excess_ was considered significant when it was at least three times as high as the standard deviation of the atom% ^15^N_control_ obtained. We assumed that the ^15^N enrichment of the animal tissue and dinoflagellates results entirely from the net assimilation of fixed N by the coral (i.e., DDN assimilation). Finally, the net N_2_ fixation by suspended particles and the DDN assimilation into the different coral compartments were calculated according to reference 81 by the equation net N_2_ fixation or DDN assimilation = (atom% ^15^N_excess_*/*[*t* × 9.8]) × (μg PN_sample_/cm^2^ or liter), where *t* is the incubation time, 9.8 is the ^15^N enrichment of the incubation water at the beginning (atom% ^15^N), and PN_sample_/cm^2^ or liter is the particulate N content of the sample at the end of the incubation normalized either per cm^2^ of coral skeletal surface area or per liter of incubation water. The skeletal surface area of the coral colonies was determined by the wax technique (82).

### Statistical analyses.

Statistical analyses were carried out with Primer-E version 6 software (83) with the PERMANOVA+ add-on (84). To look for differences in N_2_ fixation and DDN assimilation by *S. pistillata* corals exposed to different environmental conditions, one-factor PERMANOVAs based on Euclidian distance were conducted for the individual compartments (suspended particles, animal tissue, and dinoflagellates), as well as for the sum of all three compartments (total N_2_ fixation). An additional one-factor PERMANOVA was conducted to investigate differences in rates of N_2_ fixation by suspended particles after incubation of *Alveopora* species (shallow) and *S. pistillata* (shallow) corals. Type III (partial) sum of squares was used with permutation of residuals under a reduced model (999 permutations). The significance for the main test, as well for the pairwise comparisons, was based on MC tests because of the low number of unique permutations.

## References

[1] Rohwer F, Seguritan V, Azam F, Knowlton N 2002 Diversity and distribution of coral-associated bacteria. Mar Ecol Prog Ser 243:1–10. doi:10.3354/meps243001.

[2] Ainsworth TD, Thurber RV, Gates RD 2010 The future of coral reefs: a microbial perspective. Trends Ecol Evol 25:233–240. doi:10.1016/j.tree.2009.11.001. 20006405

[3] Rosenberg E, Koren O, Reshef L, Efrony R, Zilber-Rosenberg I 2007 The role of microorganisms in coral health, disease and evolution. Nat Rev Microbiol 5:355–362. doi:10.1038/nrmicro1635. 17384666

[4] Kimes NE, van Nostrand JD, Weil E, Zhou J, Morris PJ 2010 Microbial functional structure of Montastraea faveolata, an important Caribbean reef-building coral, differs between healthy and yellow-band diseased colonies. Environ Microbiol 12:541–556. doi:10.1111/j.1462-2920.2009.02113.x. 19958382

[5] Krediet CJ, Ritchie KB, Paul VJ, Teplitski M 2013 Coral-associated micro-organisms and their roles in promoting coral health and thwarting diseases. Proc R Soc B Biol Sci 280:20122328. doi:10.1098/rspb.2012.2328. PMC3574386 23363627

[6] Howarth RW 1988 Nutrient limitation of net primary production in marine ecosystems. Annu Rev Ecol Syst 19:89–110. doi:10.1146/annurev.es.19.110188.000513.

[7] Wang JT, Douglas AE 1999 Essential amino acid synthesis and nitrogen recycling in an alga-invertebrate symbiosis. Mar Biol 135:219–222. doi:10.1007/s002270050619.

[8] Tanaka Y, Grottoli AG, Matsui Y, Suzuki A, Sakai K 2015 Partitioning of nitrogen sources to algal endosymbionts of corals with long-term ^15^N-labelling and a mixing model. Ecol Modell 309–310:163–169. doi:10.1016/j.ecolmodel.2015.04.017.

[9] Ayukai T 1995 Retention of phytoplankton and planktonic microbes on coral reefs within the Great Barrier Reef, Australia. Coral Reefs 14:141–147. doi:10.1007/BF00367231.

[10] Anthony KRN, Fabricius KE 2000 Shifting roles of heterotrophy and autotrophy in coral energetics under varying turbidity. J Exp Mar Biol Ecol 252:221–253. doi:10.1016/S0022-0981(00)00237-9. 10967335

[11] Miller DJ, Yellowlees D 1989 Inorganic nitrogen uptake by symbiotic marine cnidarians: a critical review. Proc R Soc London B Biol Sci 237:109–125. doi:10.1098/rspb.1989.0040.

[12] Lema KA, Willis BL, Bourne DG 2012 Corals form characteristic associations with symbiotic nitrogen-fixing bacteria. Appl Environ Microbiol 78:3136–3144. doi:10.1128/AEM.07800-11. 22344646 PMC3346485

[13] D Ainsworth T, Krause L, Bridge T, Torda G, Raina JB, Zakrzewski M, Gates RD, Padilla-Gamiño JL, Spalding HL, Smith C, Woolsey ES, Bourne DG, Bongaerts P, Hoegh-Guldberg O, Leggat W 2015 The coral core microbiome identifies rare bacterial taxa as ubiquitous endosymbionts. ISME J 9:2261–2274. doi:10.1038/ismej.2015.39. 25885563 PMC4579478

[14] Yamamuro M, Kayanne H, Minagawao M 1995 Carbon and nitrogen stable isotopes of primary producers in coral reef ecosystems. Limnol Oceanogr 40:617–621. doi:10.4319/lo.1995.40.3.0617.

[15] Lesser MP, Falcón LI, Rodríguez-Román A, Enríquez S, Hoegh-Guldberg O, Iglesias-Prieto R 2007 Nitrogen fixation by symbiotic cyanobacteria provides a source of nitrogen for the scleractinian coral *Montastraea* *cavernosa*. Mar Ecol Prog Ser 346:143–152. doi:10.3354/meps07008.

[16] Shashar N, Cohen Y, Loya Y, Sar N 1994 Nitrogen fixation (acetylene reduction) in stony corals: evidence for coral-bacteria interactions. Mar Ecol Prog Ser 111:259–264. doi:10.3354/meps111259.

[17] Rädecker N, Meyer FW, Bednarz VN, Cardini U, Wild C 2014 Ocean acidification rapidly reduces dinitrogen fixation associated with the hermatypic coral *Seriatopora* *hystrix*. Mar Ecol Prog Ser 511:297–302. doi:10.3354/meps10912.

[18] Bednarz VN, Cardini U, van Hoytema N, Al-Rshaidat MMD, Wild C 2015 Seasonal variation in dinitrogen fixation and oxygen fluxes associated with two dominant zooxanthellate soft corals from the northern Red Sea. Mar Ecol Prog Ser 519:141–152. doi:10.3354/meps11091.

[19] Cardini U, Bednarz VN, Naumann MS, van Hoytema N, Rix L, Foster RA, Al-Rshaidat MMD, Wild C 2015 Functional significance of dinitrogen fixation in sustaining coral productivity under oligotrophic conditions. Proc Biol Sci 282:20152257. doi:10.1098/rspb.2015.2257. PMC4650168 26511052

[20] Lesser MP, Mazel CH, Gorbunov MY, Falkowski PG 2004 Discovery of symbiotic nitrogen-fixing cyanobacteria in corals. Science 305:997–1000. doi:10.1126/science.1099128. 15310901

[21] Williams WM, Viner AB, Broughton WJ 1987 Nitrogen fixation (acetylene reduction) associated with the living coral *Acropora* *variabilis*. Mar Biol 94:531–535. doi:10.1007/BF00431399.

[22] Grover R, Ferrier-Pagès C, Maguer JF, Ezzat L, Fine M 2014 Nitrogen fixation in the mucus of Red Sea corals. J Exp Biol 217:3962–3963. doi:10.1242/jeb.111591. 25278474

[23] Benavides M, Houlbrèque F, Camps M, Lorrain A, Grosso O, Bonnet S 2016 Diazotrophs: a non-negligible source of nitrogen for the tropical coral *Stylophora* *pistillata*. J Exp Biol 219:2608–2612. doi:10.1242/jeb.139451. 27335448

[24] Riemann L, Farnelid H, Steward GF 2010 Nitrogenase genes in non-cyanobacterial plankton: prevalence, diversity and regulation in marine waters. Aquat Microb Ecol 61:235–247. doi:10.3354/ame01431.

[25] Großkopf T, Mohr W, Baustian T, Schunck H, Gill D, Kuypers MMM, Lavik G, Schmitz RA, Wallace DWR, LaRoche J 2012 Doubling of marine dinitrogen-fixation rates based on direct measurements. Nature 488:361–364. doi:10.1038/nature11338. 22878720

[26] Mohr W, Grosskopf T, Wallace DW, LaRoche J 2010 Methodological underestimation of oceanic nitrogen fixation rates. PLoS One 5:e12583. doi:10.1371/journal.pone.0012583. 20838446 PMC2933240

[27] Falkowski PG, Dubinsky Z, Muscatine L, Porter JW 1984 Light and the bioenergetics of a symbiotic coral. BioScience 34:705–709. doi:10.2307/1309663.

[28] Anthony KRN, Hoogenboom MO, Maynard JA, Grottoli AG, Middlebrook R 2009 Energetics approach to predicting mortality risk from environmental stress: a case study of coral bleaching. Funct Ecol 23:539–550. doi:10.1111/j.1365-2435.2008.01531.x.

[29] Grottoli AG, Rodrigues LJ, Palardy JE 2006 Heterotrophic plasticity and resilience in bleached corals. Nature 440:1186–1189. doi:10.1038/nature04565. 16641995

[30] Cardini U, van Hoytema N, Bednarz VN, Rix L, Foster RA, Al-Rshaidat MMD, Wild C 2016 Microbial dinitrogen fixation in coral holobionts exposed to thermal stress and bleaching. Environ Microbiol 18:2620–2633. doi:10.1111/1462-2920.13385. 27234003

[31] Camps M, Benavides M, Lema KA, Bourne DG, Grosso O, Bonnet S 2016 Released coral mucus does not enhance planktonic N_2_ fixation rates. Aquat Microb Ecol 77:51–63. doi:10.3354/ame01787.

[32] Foster RA, Paytan A, Zehr JP 2009 Seasonality of N_2_ fixation and *nifH* gene diversity in the Gulf of Aqaba (Red Sea). Limnol Oceanogr 54:219–233. doi:10.4319/lo.2009.54.1.0219.

[33] Rahav E, Herut B, Mulholland M, Belkin N, Elifantz H, Berman-Frank I 2015 Heterotrophic and autotrophic contribution to dinitrogen fixation in the Gulf of Aqaba. Mar Ecol Prog Ser 522:67–77. doi:10.3354/meps11143.

[34] Bonnet S, Berthelot H, Turk-Kubo K, Fawcett S, Rahav E, L'Helguen S, Berman-Frank I 2015 Dynamics of N_s_> fixation and fate of diazotroph-derived nitrogen in a low-nutrient, low-chlorophyll ecosystem: results from the VAHINE mesocosm experiment (New Caledonia). Biogeosciences 13:2653–2673. doi:10.5194/bg-13-2653-2016.

[35] Cardini U, Bednarz VN, Foster RA, Wild C 2014 Benthic N_2_ fixation in coral reefs and the potential effects of human-induced environmental change. Ecol Evol 4:1706–1727. doi:10.1002/ece3.1050. 24967086 PMC4063469

[36] Van Duyl F, Gast G 2001 Linkage of small-scale spatial variations in DOC, inorganic nutrients and bacterioplankton growth with different coral reef water types. Aquat Microb Ecol 24:17–26. doi:10.3354/ame024017.

[37] Nakajima R, Yoshida T, Azman BAR, Zaleha K, Othman BHR, Toda T 2009 *In situ* release of coral mucus by Acropora and its influence on the heterotrophic bacteria. Aquat Ecol 43:815–823. doi:10.1007/s10452-008-9210-y.

[38] Allers E, Niesner C, Wild C, Pernthaler J 2008 Microbes enriched in seawater after addition of coral mucus. Appl Environ Microbiol 74:3274–3278. doi:10.1128/AEM.01870-07. 18344335 PMC2394949

[39] Naumann MS, Richter C, El-Zibdah M, Wild C 2009 Coral mucus as an efficient trap for picoplanktonic cyanobacteria: implications for pelagic–benthic coupling in the reef ecosystem. Mar Ecol Prog Ser 385:65–76. doi:10.3354/meps08073.

[40] Crossland CJ, Barnes DJ, Borowitzka MA 1980 Diurnal lipid and mucus production in the staghorn coral *Acropora* *acuminata*. Mar Biol 60:81–90. doi:10.1007/BF00389151.

[41] Tremblay P, Grover R, Maguer JF, Legendre L, Ferrier-Pagès C 2012 Autotrophic carbon budget in coral tissue: a new ^13^C-based model of photosynthate translocation. J Exp Biol 215:1384–1393. doi:10.1242/jeb.065201. 22442377

[42] Wild C, Huettel M, Klueter A, Kremb SG, Rasheed MY, Jørgensen BB 2004 Coral mucus functions as an energy carrier and particle trap in the reef ecosystem. Nature 428:66–70. doi:10.1038/nature02344. 14999280

[43] Fonvielle JA, Reynaud S, Jacquet S, LeBerre B, Ferrier-Pagès C 2015 First evidence of an important organic matter trophic pathway between temperate corals and pelagic microbial communities. PLoS One 10:e0139175. doi:10.1371/journal.pone.0139175. 26466126 PMC4605525

[44] Tremblay P, Weinbauer MG, Rottier C, Guérardel Y, Nozais C, Ferrier-Pagès C 2011 Mucus composition and bacterial communities associated with the tissue and skeleton of three scleractinian corals maintained under culture conditions. J Mar Biol Assoc 91:649–657. doi:10.1017/S002531541000130X.

[45] Mass T, Einbinder S, Brokovich E, Shashar N, Vago R, Erez J, Dubinsky Z 2007 Photoacclimation of *Stylophora* *pistillata* to light extremes: metabolism and calcification. Mar Ecol Prog Ser 334:93–102. doi:10.3354/meps334093.

[46] Lesser MP, Slattery M, Stat M, Ojimi M, Gates RD, Grottoli A 2010 Photoacclimatization by the coral *Montastraea* *cavernosa* in the mesophotic zone: light, food, and genetics. Ecology 91:990–1003. doi:10.1890/09-0313.1. 20462114

[47] Crossland CJ 1987 In situ release of mucus and DOC-lipid from the corals *Acropora* *variabilis* and *Stylophora* *pistillata* in different light regimes. Coral Reefs 6:35–42. doi:10.1007/BF00302210.

[48] Tremblay P, Grover R, Maguer JF, Hoogenboom M, Ferrier-Pagès C 2014 Carbon translocation from symbiont to host depends on irradiance and food availability in the tropical coral *Stylophora* *pistillata*. Coral Reefs 33:1–13. doi:10.1007/s00338-013-1100-7.

[49] Grover R, Maguer JF, Reynaud-Vaganay S, Ferrier-Pagès C 2002 Uptake of ammonium by the scleractinian coral *Stylophora* *pistillata*: effect of feeding, light, and ammonium concentrations. Limnol Oceanogr 47:782–790. doi:10.4319/lo.2002.47.3.0782.

[50] Pernice M, Meibom A, Van Den Heuvel A, Kopp C, Domart-Coulon I, Hoegh-Guldberg O, Dove S 2012 A single-cell view of ammonium assimilation in coral-dinoflagellate symbiosis. ISME J 6:1314–1324. doi:10.1038/ismej.2011.196. 22222466 PMC3379633

[51] Kopp C, Pernice M, Domart-Coulon I, Djediat C, Spangenberg JE, Alexander DTL, Hignette M, Meziane T, Meibom A 2013 Highly dynamic cellular-level response of symbiotic coral to a sudden increase in environmental nitrogen. mBio 4:e00052-13. doi:10.1128/mBio.00052-13. 23674611 PMC3656441

[52] Godinot C, Grover R, Allemand D, Ferrier-Pagès C 2011 High phosphate uptake requirements of the scleractinian coral *Stylophora* *pistillata*. J Exp Biol 214:2749–2754. doi:10.1242/jeb.054239. 21795572

[53] Mills MM, Ridame C, Davey M, La Roche J, Geider RJ 2004 Iron and phosphorus co-limit nitrogen fixation in the eastern tropical North Atlantic. Nature 429:292–294. doi:10.1038/nature02550. 15152251

[54] Al-Niemi TS, Kahn ML, McDermott TR 1997 P metabolism in the bean-Rhizobium tropici symbiosis. Plant Physiol 113:1233–1242. doi:10.1104/pp.113.4.1233. 12223671 PMC158246

[55] Batterman SA, Wurzburger N, Hedin LO 2013 Nitrogen and phosphorus interact to control tropical symbiotic N_2_ fixation: a test in *Inga punctata*. J Ecol 101:1400–1408. doi:10.1111/1365-2745.12138.

[56] Gutschick VP 1981 Evolved strategies in nitrogen acquisition by plants. Am Nat 118:607–637. doi:10.1086/283858.

[57] Israel DW 1987 Investigation of the role of phosphorus in symbiotic dinitrogen fixation. Plant Physiol 84:835–840. doi:10.1104/pp.84.3.835. 16665531 PMC1056679

[58] Gentili F, Huss-Danell K 2003 Local and systemic effects of phosphorus and nitrogen on nodulation and nodule function in *Alnus* *incana*. J Exp Bot 54:2757–2767. doi:10.1093/jxb/erg311. 14585829

[59] Robson A, O’hara G, Abbott L 1981 Involvement of phosphorus in nitrogen fixation by subterranean clover (*Trifolium* *subterraneum* L.). Funct Plant Biol 8:427–436.

[60] Karl D, Michaels A, Bergman B, Capone D, Carpenter E, Letelier R, Lipschultz F, Paerl H, Sigman D, Stal L 2002 Dinitrogen fixation in the world’s oceans, p 47–98. *In* Boyer EW, Howarth RW (ed), The nitrogen cycle at regional to global scales. Springer, Dordrecht, Netherlands.

[61] Bednarz VN, van Hoytema N, Cardini U, Naumann MS, Al-Rshaidat MMD, Wild C 2015 Dinitrogen fixation and primary productivity by carbonate and silicate reef sand communities of the northern Red Sea. Mar Ecol Prog Ser 527:47–57. doi:10.3354/meps11224.

[62] Santos HF, Carmo FL, Duarte G, Dini-Andreote F, Castro CB, Rosado AS, van Elsas JD, Peixoto RS 2014 Climate change affects key nitrogen-fixing bacterial populations on coral reefs. ISME J 8:2272–2279. doi:10.1038/ismej.2014.70. 24830827 PMC4992079

[63] Lee STM, Davy SK, Tang SL, Fan TY, Kench PS 2015 Successive shifts in the microbial community of the surface mucus layer and tissues of the coral *Acropora* *muricata* under thermal stress. FEMS Microbiol Ecol 91:289–296. doi:10.1093/femsec/fiv142. 26564958

[64] Postgate JR 1982 The fundamentals of nitrogen fixation. Cambridge University Press, Cambridge, United Kingdom.

[65] Tremblay P, Naumann MS, Sikorski S, Grover R, Ferrier-Pagès C 2012 Experimental assessment of organic carbon fluxes in the scleractinian coral *Stylophora* *pistillata* during a thermal and photo stress event. Mar Ecol Prog Ser 453:63–77. doi:10.3354/meps09640.

[66] Niggl W, Glas M, Laforsch C, Mayr C, Wild C 2009 First evidence of coral bleaching stimulating organic matter release by reef corals, p 905–911. Proc 11th Int Coral Reef Symp, 7 to 11 July 2008, Ft. Lauderdale, FL.

[67] Wooldridge SA 2009 A new conceptual model for the enhanced release of mucus in symbiotic reef corals during ‘bleaching’ conditions. Mar Ecol Prog Ser 396:145–152. doi:10.3354/meps08310.

[68] Berman-Frank I, Lundgren P, Chen YB, Küpper H, Kolber Z, Bergman B, Falkowski P 2001 Segregation of nitrogen fixation and oxygenic photosynthesis in the marine cyanobacterium Trichodesmium. Science 294:1534–1537. doi:10.1126/science.1064082. 11711677

[69] Stal LJ 2009 Is the distribution of nitrogen-fixing cyanobacteria in the oceans related to temperature? Environ Microbiol 11:1632–1645. doi:10.1111/j.1758-2229.2009.00016.x. 19397684

[70] Alamaru A, Loya Y, Brokovich E, Yam R, Shemesh A 2009 Carbon and nitrogen utilization in two species of Red Sea corals along a depth gradient: insights from stable isotope analysis of total organic material and lipids. Geochim Cosmochim Acta 73:5333–5342. doi:10.1016/j.gca.2009.06.018.

[71] Muscatine L, Kaplan I 1994 Resource partitioning by reef corals as determined from stable isotope composition II. δ^15^N of Zooxanthellae and animal tissue versus depth. Pac Sci 48:304–312.

[72] Wankel SD, Chen Y, Kendall C, Post AF, Paytan A 2010 Sources of aerosol nitrate to the Gulf of Aqaba: evidence from δ^15^N and δ^18^O of nitrate and trace metal chemistry. Mar Chem 120:90–99.

[73] Slattery M, Lesser MP, Brazeau D, Stokes MD, Leichter JJ 2011 Connectivity and stability of mesophotic coral reefs. J Exp Mar Biol Ecol 408:32–41. doi:10.1016/j.jembe.2011.07.024.

[74] Muscatine L, Porter JW, Kaplan IR 1989 Resource partitioning by reef corals as determined from stable isotope composition. Mar Biol 100:185–193. doi:10.1007/BF00391957.

[75] Grover R, Maguer J, Allemand D, Ferrier-Pagès C 2003 Nitrate uptake in the scleractinian coral *Stylophora* *pistillata*. Limnol Oceanogr 48:2266–2274. doi:10.4319/lo.2003.48.6.2266.

[76] Dubinsky Z, Jokiel PL 1994 Ratio of energy and nutrient fluxes regulates symbiosis between zooxanthellae and corals. Pac Sci 48:313–324.

[77] Ribes M, Dziallas C, Coma R, Riemann L 2015 Microbial diversity and putative diazotrophy in high- and low-microbial-abundance Mediterranean sponges. Appl Environ Microbiol 81:5683–5693. doi:10.1128/AEM.01320-15. 26070678 PMC4551227

[78] Middelburg JJ, Mueller CE, Veuger B, Larsson AI, Form A, van Oevelen D 2015 Discovery of symbiotic nitrogen fixation and chemoautotrophy in cold-water corals. Sci Rep 5:17962. doi:10.1038/srep17962. 26644069 PMC4672307

[79] Klawonn I, Lavik G, Böning P, Marchant HK, Dekaezemacker J, Mohr W, Ploug H 2015 Simple approach for the preparation of (15-15)N_2_-enriched water for nitrogen fixation assessments: evaluation, application and recommendations. Front Microbiol 6:769. doi:10.3389/fmicb.2015.00769. 26300853 PMC4523818

[80] Chen Y, Paytan A, Chase Z, Measures C, Beck AJ, Sañudo-Wilhelmy SA, Post AF 2008 Sources and fluxes of atmospheric trace elements to the Gulf of Aqaba, Red Sea. J Geophys Res 113:D05306. doi:10.1029/2007JD009110.

[81] Montoya JP, Voss M, Kähler P, Capone DG 1996 A simple, high-precision, high-sensitivity tracer assay for N_2_ fixation. Appl Environ Microbiol 62:986–993. 16535283 10.1128/aem.62.3.986-993.1996 PMC1388808

[82] Stimson J, Kinzie RA 1991 The temporal pattern and rate of release of zooxanthellae from the reef coral *Pocillopora* *damicornis* (Linnaeus) under nitrogen-enrichment and control conditions. J Exp Mar Biol Ecol 153:63–74. doi:10.1016/S0022-0981(05)80006-1.

[83] Clarke KR, Gorley RN 2006 Primer version 6: user manual/tutorial primer-E. Plymouth, England.

[84] Anderson MJ 2001 A new method for non-parametric multivariate analysis of variance. Austral Ecol 26:32–46. doi:10.1046/j.1442-9993.2001.01070.x.

